# Improving teaching on an inpatient pediatrics service: a retrospective analysis of a program change

**DOI:** 10.1186/1472-6920-12-92

**Published:** 2012-10-01

**Authors:** Michael A Barone, Robert A Dudas, Rosalyn W Stewart, Julia A McMillan, George J Dover, Janet R Serwint

**Affiliations:** 1 Johns Hopkins University School of Medicine, BRB 137 733 North Broadway, Baltimore, MD, 21205, USA

**Keywords:** Medical education, Teaching attending, Clinical skills

## Abstract

**Background:**

The traditional role of the faculty inpatient attending providing clinical care and effectively teaching residents and medical students is threatened by increasing documentation requirements, pressures to increase clinical productivity, and insufficient funding available for medical education. In order to sustain and improve clinical education on a general pediatric inpatient service, we instituted a comprehensive program change. Our program consisted of creating detailed job descriptions, setting clear expectations, and providing salary support for faculty inpatient attending physicians serving in clinical and educational roles. This study was aimed at assessing the impact of this program change on the learners’ perceptions of their faculty attending physicians and learners’ experiences on the inpatient rotations.

**Methods:**

We analyzed resident and medical student electronic evaluations of both clinical and teaching faculty attending physician characteristics, as well as resident evaluations of an inpatient rotation experience. We compared the proportions of “superior” ratings versus all other ratings prior to the educational intervention (2005–2006, baseline) with the two subsequent years post intervention (2006–2007, year 1; 2007–2008, year 2). We also compared medical student scores on a comprehensive National Board of Medical Examiners clinical subject examination pre and post intervention.

**Results:**

When compared to the baseline year, pediatric residents were more likely to rate as superior the quality of didactic teaching (OR=1.7 [1.0-2.8] year 1; OR=2.0 [1.1-3.5] year 2) and attendings’ appeal as a role model (OR=1.9 [1.1-3.3] year 2). Residents were also more likely to rate as superior the quality of feedback and evaluation they received from the attending (OR=2.1 [1.2-3.7] year 1; OR=3.9 [2.2-7.1] year 2). Similar improvements were also noted in medical student evaluations of faculty attendings. Most notably, medical students were significantly more likely to rate feedback on their data gathering and physical examination skills as superior (OR=4.2 [2.0-8.6] year 1; OR=6.4 [3.0-13.6] year 2).

**Conclusions:**

A comprehensive program which includes clear role descriptions along with faculty expectations, as well as salary support for faculty in clinical and educational roles, can improve resident and medical student experiences on a general pediatric inpatient service. The authors provide sufficient detail to replicate this program to other settings.

## Background

In many academic medical centers individual generalist and subspecialty faculty members contribute to the effort of caring for general inpatients. General inpatients are those patients who are not admitted to the service of their primary care provider or to a subspecialist, either because of those physicians’ lack of admitting privileges or because the patient’s diagnosis is not restricted to a single organ system. The role of caring for such inpatients is often referred to as “ward attending,” “service attending” or “clinical attending” and typically includes the teaching of residents and medical students as a major responsibility [1,2]. This traditional model is increasingly being challenged as a result of diminished dollars directed towards education, pressure to increase clinical productivity and increasing documentation requirements for patient care [3,4]. Pediatric departments have reported difficulty securing faculty for teaching duties, while at the same time standards for medical education are expanding to include increased emphasis on direct observation of trainees and timely feedback and assessment of residents and students [5,6]. These increasing demands have created a situation wherein one faculty member may not be able to meet the expectations of patient care and teaching on a clinical service.

In this paper, we describe the implementation and evaluation of a multifaceted model for the clinical care and educational coverage of a general inpatient pediatrics service in a large, urban academic pediatrics department. Our system is distinct from the more recent trend to provide inpatient coverage through employing a team of hospitalists [7]. Our faculty represent numerous generalist and specialty divisions within our department and each faculty member has his or her additional area of academic pursuit which sustains them in a single promotions track environment. In seeking to evaluate the effectiveness of our educational intervention, we hypothesized that financial support and a defined job description would lead to improved resident and medical student evaluations of faculty attendings, specifically in the domains of feedback given to trainees on their communication, data gathering and physical exam skills. Previous investigators have demonstrated the association between paying faculty members for teaching and improved educational experiences for learners [8]. We chose to focus on the outcome of trainee evaluations since these have been shown to correlate with multiple indicators of effective teaching [9]. As secondary outcomes, we compared 1) resident evaluation of the ward rotation experience and 2) medical student content knowledge as assessed by the National Board of Medical Examiners clinical subject examination before and after modifying the attending system.

### Original program

The Department of Pediatrics at the Johns Hopkins University School of Medicine has approximately 150 full-time faculty members who are Board certified pediatricians in its main facility, the Johns Hopkins Children’s Center. General inpatients are cared for by two resident teams with medical students assigned to each team. In 1996, the Chairman of the Department of Pediatrics created an additional faculty position – the teaching attending. One teaching attending would serve on each of the two inpatient teams along with a ward attending for each of the two teams. The driving forces behind this decision were increasing patient volume and acuity, increasing requirements for documentation and billing, and concerns that medical student teaching was no longer a priority. The teaching attending’s primary responsibility was to focus on medical student education. Faculty volunteered for this teaching attending position but rarely reduced their other responsibilities, as no additional funding came with the position to allow for dedicated time. The list of responsibilities was loosely interpreted and difficult to enforce due to the volunteer nature of the position. As a result, faculty would often serve in the role for time periods that were briefer than the students’ inpatient rotation, leading students to have several teaching attendings during the course of their rotation. This limited the extent to which any one faculty member was able to teach and assess a student.

Concurrently, similar concerns were being raised by the pediatrics housestaff that the clinical ward attending was becoming less available for teaching and patient care. Increasing productivity demands often required the attending to be off the inpatient units in outpatient clinics. Volunteer ward attendings, like the teaching attendings, offered to serve for only one to two weeks at a time, diminishing continuity for both patient care and resident assessment. Decreasing ward attending presence on the inpatient units also ran counter to a patient safety movement that was spreading across the institution [10]. While serving in this role had previously been considered an honor, over time it became increasingly difficult to have faculty volunteer for a ward attending rotation. As a result, we were threatened with unfilled schedules and impairments to the traditional quality of our inpatient teaching rotations.

### Creation of a new program

In July of 2006, the department chairman, residency program directors, and the clerkship director redefined the positions of the clinical ward attending and the medical student teaching attending. The goals of the new program were to 1) improve medical education, 2) create a system of accountability for teaching efforts - linked to funding, and 3) begin to nurture a cohort of generalist and specialty faculty interested in medical education and associated faculty development [11,12]. In attempting to achieve these goals, we proceeded with the following guiding principles; 1) the clinical ward attending would focus primarily on clinical care of the patients and resident education, 2) the teaching attending would focus on inpatient medical student education, 3) although attending presence would increase, all efforts would be made to maintain resident autonomy, a key feature in our residency training program and 4) faculty development would enable faculty to fulfill either position throughout the academic year and would support a community of educators interested in continuous improvement.

The expectations of the clinical ward attending and the medical student teaching attending are shown in Tables 1 and 2 respectively. The specific responsibilities emphasize the provision of outstanding patient care, resident education and feedback and direct observation of the student in their clinical role with patients. Faculty serving as ward attendings are required to devote 90% of their time, during their rotation, to the position and those serving as teaching attending are required to spend 50% of their time, during their rotation, on the assignment. In order to maintain a learner centric approach, those in either role are expected to commit to the entire rotation, mirroring the student academic calendar (4.5 weeks) or the resident monthly calendar (4 weeks). Individual salary support from the Department of Pediatrics is given to the ward attending and medical student teaching attending. Originally, one-third of the funds to pay for faculty effort came from a redistribution of departmental general funds. We also sought funding from the School of Medicine, noting that we could provide an increase in faculty-medical student contact teaching hours and improved performance in direct observation and feedback. This was particularly relevant in the setting of an upcoming review by the Liaison Committee on Medical Education. We also sought funding from the Johns Hopkins Hospital. The hospital did not agree to provide funding but the medical school did. The Department of Pediatrics set salary allocation as 0.08 FTE for 4 weeks of ward attending and 0.05 FTE for 4.5 weeks of teaching attending. When the above changes were announced to begin in July 2006, we noted a significant increase in the number of faculty willing to serve as ward and teaching attending. We were able to select individuals for ward and teaching attending roles based on past teaching experience and excellence.

**Table 1:** Expectations of ward attending*

**Responsibility**	**Time Commitment**
Review of goals and objectives with ward team	30 min/month
Attending rounds with entire team	3 hr/week
Morning Report attendance	3 hr/week
Bedside teaching	2 hr/week
Observation of daily work rounds	1.5 hr/week
Clinical care of patients	21 hr/week
Direct observation and feedback of interns	4 hr/month
Review and feedback of intern write-ups	2 hr/month
Facilitate journal club	2 hr/month
Mid-rotation feedback to residents	2 hr/month
End of rotation feedback to residents	2 hr/month
Completion of evaluation forms	1 hr/month
Attend faculty development meetings	1 hr/month

*Two interns, 2 senior residents and subintern on service.

**Table 2:** Expectations of Medical Student Teaching Attending*

**Responsibility**	**Time Commitment**
Didactic teaching sessions with medical students	4 hr/week
Direct observation and feedback of medical students	4 hr/week
Read, give feedback on medical student write-ups	3 hr/week
Attending rounds with entire team	3 hr/week
Observation of daily work rounds	3 hr/week
Facilitate Journal club	2 hr/month
Mid-rotation feedback to students	2 hr/month
End of rotation feedback to students	2 hr/month
Completion of evaluation forms	1 hr/month
Attend faculty development meetings	1 hr/month

*Four medical students rotate on each service.

## Methods

Evaluation outcomes for both groups included the deidentified resident and medical student responses to electronic evaluations (E*value®) completed at the end of their rotation. We selected a pre-post study design by comparing evaluations from the academic year preceding the development of our program (2005–2006) with the two subsequent academic years (2006–2007, 2007–2008). Two subsequent years were studied to determine sustainability of the program effects. Scoring was done on a 1 (Poor) -5 (Superior) Likert Scale with descriptive anchors. Neither the residents nor medical students were aware that the aggregate data was being studied. The study was approved by the Institutional Review Board of the Johns Hopkins University School of Medicine.

We compared evaluations of the ward attendings qualities (15 questions-Table 3) and the general inpatient rotation (13 questions - Table 4) as rated by the pediatric residents. We also analyzed evaluations of the teaching attendings (13 questions - Table 5) as rated by the medical students. Open field narrative comments were encouraged on all evaluations. We also compared end of clerkship National Board of Medical Examiners clinical subject examination in Pediatrics before and after the attending system was modified.

**Table 3:** **Resident evaluation of ward attending**

**Superior (score of 5) vs. other**								
**N evaluations**	**2005-2006**		**2006-2007**		**Odds Ratio 05–06 vs 06-07**	**2007-2008**		**Odds Ratio 05–06 vs 07-08**
	**120**		**144**			**116**		
	**#**	**%**	**#**	**%**		**#**	**%**	
Quality of Bedside Teaching	42	35	58	40	1.3 (0.7-2.1)	59	51	1.9 (1.1-3.4)
Assistance with administrative/social problems	41	34	60	42	1.4 (0.8-2.3)	63	54	2.3 (1.3-4.0)
Sub-Intern Involvement	46	38	49	34	0.8 (0.5-1.4)	39	34	0.8 (0.5-1.4)
Delegation of Responsibility	55	46	68	47	1.0 (0.6-1.7)	66	57	1.6 (0.9-2.7)
Stimulates Critical Thinking	63	53	85	59	1.3 (0.8-2.2)	74	64	1.6 (0.9-2.8)
Quality of Didactic Teaching	53	44	82	57	1.7 (1.0-2.8)	71	61	2.0 (1.1-3.5)
Clarity of Expectations	49	41	71	49	1.4 (0.8-2.4)	67	58	2.0 (1.1-3.4)
Availability	65	54	86	60	1.3 (0.8-2.1)	75	65	1.5 (0.9-2.7)
Approachability	80	67	103	72	1.2 (0.7-2.2)	90	78	1.7 (0.9-3.2)
Teaching Commitment	74	62	96	67	1.2 (0.7-2.1)	82	71	1.5 (0.8-2.7)
Feedback/Evaluation	28	23	56	39	2.1 (1.2-3.7)	63	54	3.9 (2.2-7.1)
Professionalism	65	54	98	68	1.8 (1.1-3.1)	79	68	1.8 (1.0-3.2)
Communication with the team	56	47	72	50	1.1 (0.7-1.9)	68	59	1.6 (0.9-2.8)
Timeliness	49	41	73	51	1.5 (0.9-2.5)	65	56	1.8 (1.1-3.2)
Appeal as a Role Model	63	53	89	62	1.5 (0.9-2.5)	78	67	1.9 (1.1-3.3)

**Table 4:** **Resident evaluation of ward rotation**

**Superior (score of 5) vs. other**								
**N evaluations**	**2005-2006**		**2006-2007**		**Odds Ratio 05–06 vs 06-07**	**2007-2008**		**Odds Ratio 05–06 vs 07-08**
	**92**		**101**			**89**		
	**#**	**%**	**#**	**%**		**#**	**%**	
Attending Availability	48	52	59	58	1.3 (0.7 - 2.3)	53	60	1.3 (0.8 - 2.4)
Educational Interaction with attendings/fellows	43	47	50	50	1.1 (0.6 - 2.0)	48	54	1.3 (0.7 - 2.4)
Lectures conferences quality	25	27	39	39	1.7 (0.9 - 3.1)	41	46	2.3 (1.2 - 4.3)
Patient related teaching quality	35	38	44	44	1.3 (0.7 - 2.3)	39	44	1.3 (0.7 - 2.3)
Support from other residents	35	38	45	45	1.3 (0.7 - 2.3)	36	40	1.1 (0.6 - 2.0)
Appropriateness of independence	55	60	61	60	1.0 (0.6 - 1.8)	50	56	0.9 (0.5 - 1.6)
Involvement as team member	63	68	65	64	0.8 (0.5 - 1.5)	55	62	0.7 (0.4 - 1.4)
Experience with Procedures	5	5	8	8	1.5 (0.5 - 4.8)	6	7	1.3 (0.4 - 4.3)
Overall quality of rotation	41	45	54	53	1.4 (0.8 - 2.5)	37	42	0.9 (0.5 - 1.6)

**Table 5:** **Med student evaluation of teaching attending**

**Superior (score of 5) vs. other**								
**N**	**2005-2006**		**2006-2007**		**Odds Ratio 05–06 vs 06-07**	**2007-2008**		**Odds Ratio 05–06 vs 07-08**
	**80**		**78**			**75**		
	**#**	**%**	**#**	**%**		**#**	**%**	
Medical Knowledge	68	85	66	85	1.0 (0.4-2.6)	67	89	1.5 (0.5-4.4)
Stimulates Critical Thinking	58	73	66	85	2.1 (0.9-5.0)	66	88	2.8 (1.1-7.4)
Quality of Didactic sessions	49	61	67	86	3.9 (1.7-9.3)	60	80	2.5 (1.2-5.6)
Clarity of Expectations	51	64	62	79	2.2 (1.0-4.8)	55	73	1.6 (0.7-3.3)
Availability	50	63	62	79	2.3 (1.1-5.1)	62	83	2.9 (1.3-6.6)
Approachability	62	78	73	94	4.2(1.4-15.3)	66	88	2.1 (0.8-5.8)
Teaching Commitment	66	83	74	95	3.9 (1.1-17.0)	71	95	3.8 (1.1-16.4)
Feedback/Evaluation (Written/Verbal Skills)	36	45	62	79	4.7 (2.2-10.3)	64	85	7.1 (3.1-17.0)
Feedback/Evaluation (Data Gathering/Physical Exam)	23	29	49	63	4.2 (2.0-8.6)	54	72	6.4 (3.0-13.6)
Communication with the team	48	60	68	87	4.5 (1.9-11.2)	61	81	2.9 (1.3-6.5)
Timeliness	55	69	64	82	2.1 (0.9-4.7)	54	72	1.2 (0.6-2.5)
Appeal as a Role Model	61	76	68	87	2.1 (0.9-5.5)	66	88	2.3 (0.9-6.2)
Quality of Bedside teaching	33	41	57	73	3.9 (1.9-8.0)	52	69	3.2 (1.6-6.6)

For all evaluation comparisons, the proportion of residents and students scoring a question as “superior” (Likert scale = 5) was compared to an aggregate of other responses (Likert scale 1–4). We adopted this method considering that any response other than the highest possible was less than ideal. This approach has been described elsewhere and eliminates the need to convert ordinal responses to mean scores [13]. We calculated odds ratios and 95%CI in order to assess significance of any changes. National Board of Medical Examiners Clinical Subject Examination scores were compared using student t-tests. Data analysis was performed with the use of Stata, version 9.2 (StataCorp LP, College Station, Tex). This study had no extramural support.

## Results

Compared with needing more than 33 individual attendings to cover 46 necessary ward and teaching attending schedule blocks in 2005–2006, the addition of a defined role and faculty salary support enabled us to fill the ward and teaching attending schedule with an average of 26 faculty members per year from 2006–2008. This smaller group of faculty members includes individuals in general pediatrics, emergency medicine, medicine-pediatrics, as well as many pediatric subspecialties such as cardiology, genetics, hematology, infectious diseases and endocrinology.

### Resident ward attending program

The resident evaluations of ward attendings are reported in Table 3. One hundred and twenty evaluations were collected in the academic year prior to restructuring. In the subsequent years, 144 and 116 evaluations were collected respectively. For each year of the study, we collected ≥ 90% of the evaluations distributed to residents. In the year immediately following the restructured role and salary support for the ward attending, significant improvements were noted in the residents’ perception of the quality of feedback and evaluation (OR=2.1 [1.2-3.7]) as well as the perception of the professionalism of the ward attending. (OR=1.8 [1.1-3.1]). A positive trend was seen in the residents’ perception of the quality of didactic teaching (OR=1.7 [1.0-2.8] p=0.04). In the subsequent academic year (2007–2008), the positive changes noted in the first year remained and additional findings demonstrated significance. Compared to the baseline year, residents were approximately twice as likely to rate as superior (Score of 5) the attending’s bedside teaching, help in sorting out administrative and social problems, clarity of expectations, didactic teaching quality, timeliness, and appeal as a role model. Residents were nearly 4 times as likely to rate the attendings’ feedback to and evaluation of learners as superior (OR=3.9 [2.2-7.1]).

When comparing the resident evaluation of the ward rotation experience in the baseline year to the two subsequent academic years (Table 4), we noted a trend toward improved quality of lectures and conferences in 2006–2007 and a significant change in the 2007–2008 academic year (OR=2.3 [1.2-4.3]). Other parameters of the evaluation showed no significant changes.

### Medical student teaching attending program

Eighty teaching attending evaluations were collected in 2005–2006 compared to 78 in 2006–2007 and 75 in 2007–2008). The lowest evaluation completion rate by year was 94%. In the year following the restructured role and salary support for the teaching attending (2006–2007), significant improvements were noted in 8 of the 13 evaluation domains (Table 5). Some of the largest effects were seen in the students’ perception of the quality of feedback on the learners’ oral and written communication (OR=4.7 [2.2-10.3]) and history taking and physical exam skills (OR=4.2 [2–8.6]). Additional positive effects were seen in the students’ perception of the attendings’ didactic session quality, bedside teaching quality, availability, approachability, teaching commitment, and communication. In our effort to evaluate the sustainability of these findings over an additional academic year, we noted even greater improvements in some areas when comparing the 2007–2008 academic year to the baseline 2005–2006 year. Medical students were 6–7 times more likely to rate feedback on their data gathering, physical exam, oral and written communication skills as superior. Students were also more likely to rate as superior the teaching attendings’ availability (OR=2.9 [1.3-6.6]). There were no significant improvements seen in the perception of attending medical knowledge or the attending physician’s appeal as a role model.

Review of the narrative comments on teaching attending evaluations noted a number of themes. These included significant differences in attending availability compared to other clerkships, overall quality of the teaching, and the fact that students and attendings were able to get to know one another as individuals. Some representative comments included:

"“I was impressed withmy teaching attending’s earnestdesire to know eachstudent on the teampersonally. Because of thetrusting relationship that developed,he has great powerto teach effectively andoffer constructive feedback.”"

"“The teaching attending set-upwas GREAT and Iloved having meetings multipletimes every week andlearning about general pedstopics. He was somuch more available thanthe clinical attending.”"

"“The teaching attending sessionswere invaluable in givingus time to discussour patients and havebedside teaching.”"

Despite improved student perception of their educational experience, as measured by teaching attending evaluations, NBME clinical subject examination scores in pediatrics did not differ when comparing the 2005–2006 academic year with subsequent academic years. The mean score range over the years of analysis was 76.9-77.8. (p>0.05 for all comparisons- data not shown).

## Discussion

A comprehensive redesign of a pediatric inpatient teaching service which included a formal job description, a significant focus on resident and medical student education, and salary support, led to improvements in multiple educator performance ratings. We have demonstrated improvements in feedback and evaluation of both residents and medical students. In addition, students and residents ratings of the quality of bedside and didactic teaching was more likely to be considered “superior.” We achieved these positive changes in our teaching program through the thoughtful development of defined roles and expectations for faculty in each attending role, as well as offering salary support for accountability for teaching efforts. By doing so, we have been able to move our teaching program forward in many ways. Our inpatient attending coverage has continued to evolve into a smaller group of more dedicated faculty – with an average of 23 faculty per year required to fill all the medical student teaching attending and ward attending responsibilities in the 2008–2011 academic years. The design of our service continues today and remains highly successful in terms of providing clinical care and education to residents and medical students.

While hospitalist systems have also been able to demonstrate effective teaching, our system is a different clinical care model. Our faculty group represents both generalist and subspecialty faculty attendings, all of whom have other aspects to their career, such as ambulatory care and subspecialty clinical care, research, educational administration, clinical program building, and teaching [14-17]. Our institution currently does not have an academic division of hospital medicine. Our current model hybridizes the benefits of a traditional ward attending service, which includes enhancing resident autonomy and ownership in decision making, along with participation of faculty from subspecialty and general pediatrics, while including additional hours of dedicated attending time for teaching and clinical care. Our hybrid model allows individual faculty to contribute part of their overall effort to the general service yet maintain their home in their academic division. We facilitate this community of ward and teaching attending faculty through faculty development sessions throughout the year on such topics as giving and receiving feedback, small group teaching and improving bedside teaching.

Our program change has also allowed us to achieve important increases in the residents’ and students’ perceptions of the characteristics of the best clinical teachers previously documented in the literature. These include professionalism, punctuality/timeliness, communication and the conveying of clear expectations. The extent of the combined teaching and clinical roles of our faculty ward attendings allows for sufficient time with residents, thus providing a uniform exposure to enhance role modeling [18,19]. This may help to shape the eventual teaching skills and career choices of our residents and medical students.

To our knowledge this is the first formal report of wide scale implementation of a teaching attending program for medical students on an inpatient service. Previous brief reports have demonstrated the value of dedicated faculty shifts focused on medical student and resident teaching in an academic emergency department [20]. As a result of this change, we have documented significant changes in the students’ perception of the quality of bedside teaching. Our students, like others, continue to seek more direct observation of their skills, and faculty continue to struggle with challenges of dedicating the substantial amounts of time needed to view entire patient workups or to pre-round with medical students. The medical student teaching attending program has been able to reverse some of the declines in bedside teaching that have been documented in the literature over the past decades [21-23].

Strengths of our analysis include the anonymous evaluation system assessing multiple domains and our high evaluation response rate. Student and resident anonymity in our evaluation system limited any concerns that learners would be wary of retribution for criticism. Furthermore, students and residents were unaware that this program was being studied. We assessed the program over two years in order to show sustainability of our findings. In fact, for important domains such as feedback, there was a stronger impact in the second evaluation year compared to the first.

Some study limitations exist. With the exception of the analysis of the medical students’ National Board of Medical Examination clinical subject exam scores, our E*value survey instruments have not been validated. Another possible limitation is the possibility that medical student ratings of their residents and attending teachers may be subject to temporal improvements. To address this, we analyzed whether resident teachers were rated more favorably by medical students over two subsequent years with the new teaching attending program in place. No significant changes were noted in medical student evaluations of the resident teachers during this time (data not shown).

Finally, it’s conceivable that faculty evaluations may have improved on the basis of which faculty members were selected as teaching faculty for this redesigned service. We did not, however, see changes in all of the evaluation domains as may have been expected with an extremely popular and skillful set of teachers. Despite this potential limitation, our department has always approached the changes to the inpatient services as that of a multi-faceted program. This is to say that, without this intervention, we would not have been able to recruit and retain quality faculty teachers. The elements of the program such as clear expectations and salary support, along with faculty development sessions, have enabled us to achieve our goal of significantly improving our teaching program.

Our challenges relate to ongoing funding of this educational program in a tentative environment. Given the reality of low professional fee reimbursement for the clinical activities of the ward attending in Maryland, this service is not able to fully cover the 3.0 full-time equivalent salary needs. While teaching attending activities are not billable, our ongoing analysis of ward attending clinical collections over the last full academic year (2011–2012) demonstrates an offset of approximately 65% of the total salary costs of this program. As a result of this, continued departmental funding and funding from the School of Medicine is necessary. Given the current financial environment, this funding may be threatened in the future and such a system may need to demonstrate more objective educational outcomes and/or changes which enhance clinical efficiency and patient safety in order to gain wide support.

## Conclusions

Through the provision of salary support and a defined role description and clear expectations, we have been able to create a sustainable model of attending coverage on a large, academic pediatric general inpatient service comprised of generalist and subspecialty faculty. In doing so, we have demonstrated improved perception of faculty teachers by residents and medical students.

## Competing interests

The authors declare that they have no competing interests.

## Authors’ contributions

MAB, JAM, GJD and JRS designed and funded the educational program intervention. RAD and JRS assisted MAB in the design, data collection and statistical analysis of the project. RAD, RS, JAM, GJD and JRS assisted MAB in the interpretation of the results, the drafting of the manuscript, and critically evaluating earlier drafts of the manuscript. All authors read and approved the final manuscript.

## Authors’ information

MAB is associate professor and director of medical student education, Department of Pediatrics, Johns Hopkins University School of Medicine. He is President-Elect of the Council on Medical Student Education in Pediatrics (COMSEP).

RAD is assistant professor and associate director of the pediatric clerkship, Department of Pediatrics, Johns Hopkins University School of Medicine. He is also a leader of the Helen Taussig College in the School of Medicine and director of the division of hospitalist medicine at the Johns Hopkins Bayview Medical Center.

WS is associate professor, Departments of Medicine and Pediatrics, Johns Hopkins University School of Medicine. She is also associate residency Program director of the medicine - pediatrics urban health residency program at Johns Hopkins.

JAM is professor and executive vice chair, Department of Pediatrics. She is associate dean for graduate medical education, Johns Hopkins University School of Medicine

GJD is chairman, Department of Pediatrics, Johns Hopkins University School of Medicine

JRS is pediatric residency program director and professor of pediatrics and public health at the Johns Hopkins University School of Medicine and the Johns Hopkins Bloomberg School of Public Health. Department of Pediatrics, Johns Hopkins University School of Medicine. 

## Ethical approval

This analysis was considered exempt by the Johns Hopkins University Institutional Review Board.

## Previous presentations

Portions of this work were presented at the 2008 Council on Medical Student Education in Pediatrics (COMSEP) meeting and the 2009 Pediatric Academic Societies (PAS) meeting.

## Pre-publication history

The pre-publication history for this paper can be accessed here:

http://www.biomedcentral.com/1472-6920/12/92/prepub
